# miR-18a Impairs DNA Damage Response through Downregulation of Ataxia Telangiectasia Mutated (ATM) Kinase

**DOI:** 10.1371/journal.pone.0025454

**Published:** 2011-09-27

**Authors:** Libing Song, Chuyong Lin, Zhiqiang Wu, Hui Gong, Yong Zeng, Jueheng Wu, Mengfeng Li, Jun Li

**Affiliations:** 1 State Key Laboratory of Oncology in Southern China, Department of Experimental Research, Cancer Center, Sun Yat-sen University, Guangzhou, Guangdong, China; 2 Department of Biochemistry, Zhongshan School of Medicine, Sun Yat-sen University, Guangzhou, Guangdong, China; 3 Department of Microbiology, Zhongshan School of Medicine, Sun Yat-sen University, Guangzhou, Guangdong, China; Istituto Dermopatico dell'Immacolata, Italy

## Abstract

The DNA damage response (DDR) encompasses multi-step processes by which cells evolve to sense DNA damage, transduce the signal and initiate the repair of damaged DNA. Ataxia Telangiectasia Mutated (ATM) Kinase, which functions as the primary sensor and transducer of DNA damage signal, has been demonstrated to play an important role in the DDR and cancer prevention. Hence, understanding the molecular mechanisms underlying the regulation of ATM has received much attention. Here, we found that miR-18a was upregulated in both cell lines and patients' tissue samples of breast cancer. Furthermore, we demonstrated that ectopically expressing miR-18a downregulated ATM expression by directly targeting the ATM-3′-UTR and abrogated the IR-induced cell cycle arrest. Similar to the effect of ATM siRNA, overexpressing miR-18a in breast cancer cells reduced the DNA damage repair ability and the efficiency of homologous recombination-based DNA repair (HRR) and sensitized cells to γ-irradiation (IR) treatment. However, inhibition of miR-18a led to augmentation of DNA damage repair, increase of HRR efficiency and reduced cellular radiosensitivity. Moreover, we showed that the phorsphorylation level and nuclear foci formation of H2AX and 53BP1, the downstream substrates of ATM kinase, were significantly deceased in miR-18a overexpressing cells. Taken together, our results uncover a new regulatory mechanism of ATM expression and suggest that miR-18a might be a novel therapeutic target.

## Introduction

DNA damage response, an intricate signal transduction pathway, responds to DNA damage by initiating DNA repair or inducing apoptosis [1]. Amount of evidence has demonstrated that defects in any of the DDR related genes, which are responsible for sensing the damage, signal transduction and repairing double strand breaks (DSBs), could result in chromosomal breaks, fusions, and translocations, that ultimately to tumorigenesis. For instance, DDR related genes, such as RFC5, DPB11, MEC1, DDC2, MEC3, RAD53, CHK1, PDS1, and DUN1, have been reported to play important role in the maintenance of genomic stability and dysregulations of these genes were frequently found in many cancer types [2]–[5].

ATM (ataxia-telangiectasia mutated), a serine/threonine protein kinase, functions as a transducer of the DNA damage signal to the downstream effector machinery. In response to DSBs induction, ATM protein kinase becomes an active monomer from inactive dimer in a process involving autophosphorylation on serine (Ser^1981^) [6]. The active ATM rapidly hyperphosphorylates and activates downstream effecter CHK2 [7]. Furthermore, the activated CHK2 kinase phosphorylates its key substrates, CDC25A and CDC25C, which results in cells being arrested in the S phase or G2/M phases, respectively [8]. Meanwhile, ATM also hyperphosphorylates H2AX and p53 binding protein 1 (53BP1), upon DNA damage, which then form discrete nuclear foci at the site of the damaged DNA, which could amplify the degree of DDR [9]–[10]. The phosphorylated H2AX (γ-H2AX), one of the earliest ATM-dependent responses to IR, concentrated on the DNA lesions and formed discrete nuclear foci with phosphorylated 53BP1 protein, which was critical for recruiting various DNA repair proteins, such as BRCA1, NBS1, Rad51 and TopoBP1 [11]–[13]. ATM deficient cells showed no hyperphosphorylation and reduced foci formation of γ-H2AX and 53BP1 in response to IR compared with cells expressing wild-type ATM, resulting low DNA repair efficiency [9], [14].

ATM was named for the autosomal recessive progressive neurodegenerative disease, ataxia-telangiectasia (AT), which is caused by mutations of ATM gene [15]. The clinical symptoms associated with loss of ATM activity are neurodegeneration, extreme cellular sensitivity to radiation, immunodeficiency, and a predisposition to cancer [16]. Constantly, ATM deficient mice displayed growth retardation, neurologic dysfunction, radiosensitivity, sterility, defects in T lymphocyte maturation and predisposition to cancers with increased levels of hematopoietic malignancies in particular [17]. These phenotypic manifestations in both AT patients and ATM-deficient mice demonstrate that the pleiotropic function of ATM kinase is associated with various biological processes, including DNA repair, G1/S, intra-S and G2/M checkpoints, apoptosis, translation initiation, gene regulation and telomere maintenance [18]. Consistent with its function, mutations within *ATM* or repression of ATM have been found in multiple cancer types, such as breast cancer, pancreatic cancer, myeloma, leukemias and lymphomas [19]–[24]. Hence, understanding the molecular mechanisms underlying the regulation of ATM in cancers has received much attention.

Analysis with two publicly available algorithms (TargetScan and miRanda), we found that ATM protein kinase is theoretically the target gene of miR-18a. Furthermore, we demonstrated that overexpression of miR-18a downregulated ATM expression by directly targeting the ATM-3′-UTR, consequently resulting in reduction of the DNA damage repair ability and the HRR efficiency, and an increase in cellular radiosensitivity to IR treatment. Taken together, these results suggest that miR-18a plays an important role in the regulation of ATM and may represent a therapeutic target for cancers and other diseases.

## Results

### miR-18a Is Overexpressed in Breast Cancer Cell Lines and Breast Cancer Tissues

**Figure 1:**
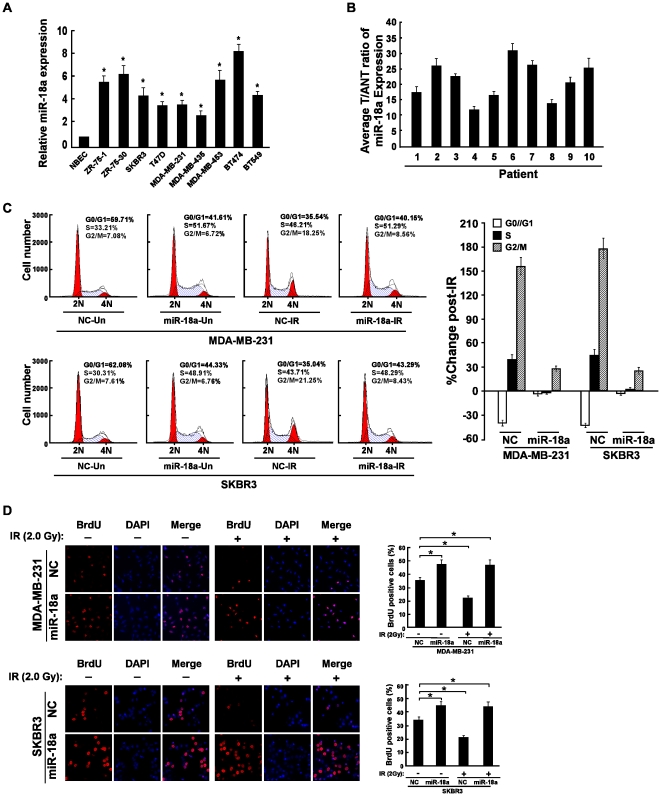
Upregulation of miR-18a abrogated IR-induced cell cycle arrest. **A,** Real-time PCR analysis of miR-18a expression in normal breast epithelial cells (NBEC) and breast cancer cell lines, including ZR-75-1, ZR-75-30, SKBR3, T47D, MDA-MB-231, MDA-MB-435, MDA-MB-453, BT474 and BT-549. **B**, The expression of miR-18a was examined in 10 paired breast tumor tissues (tumor) and their adjacent normal tissues (normal). The average miR-18a expression was normalized by U6 expression. Each bar represents the mean of three independent experiments. **C,** Flow cytometric analysis of indicated breast cancer cells transduced with miR-18a mimic or NC, treated with or without 2.0 Gy IR. Statistic analysis revealed the proportion change of cells at each phase of cell cycle from three independent experiments under different miR-18a expression levels with or without IR treatment, using the algorithm (S^+IR^−S^-IR^)/S^-IR^×100%. **D,** Representative micrographs (left) and quantification analysis (right) of BrdUrd incorporating-cells in indicated cells with or without 2.0 Gy IR.

Real-time PCR analyses revealed that miR-18a was significantly overexpressed in all of the 9 examined breast cancer cell lines, including ZR-75-1, ZR-75-30, SKBR3, T47D, MDA-MB-231, MDA-MB-435, MDA-MB-453, BT474 and BT-549, as compared with that in normal breast epithelial cells (NBEC) (Figure 1A). Furthermore, comparative analysis revealed that miR-18a was differentially overexpressed in 10 examined tissue samples paired with adjacent non-cancerous tissues from the same patient (Figure 1B), indicating that miR-18a is generally upregulated in breast cancer.

### miR-18a Regulates DNA Damage-induced Cell Cycle Checkpoint

To investigate the biological role of miR-18a on the development and progression of breast cancer, the impact of miR-18a on proliferation of breast cancer cells was evaluated using flow cytometry. As shown in Figure 1C, ectopic expression of miR-18a dramatically decreased the proportion of cells in the G1/G0 peak and increased the proportion of cells in the S peak in both breast cancer cells MDA-MB-231 and SKBR3 as compared with that of negative control (NC) transfected cells. Furthermore, BrdUrd incorporation assay was performed and showed that the S-phase fraction with BrdUrd incorporation dramatically increased in MDA-MB-231(47.5%) and SKBR3 (44.7%) cells ectopically expressing miR-18a, as compared with the NC-transected control cells (36.2% and 33.7% for MDA-MB-231 and SKBR3 control cells, respectively (Figure 1D), suggesting that miR-18a is involved in the regulation of cell cycle.

Strikingly, we found that IR-induced cell cycle arrest in MDA-MB-231 and SKBR3 cells were also abolished by miR-18a overexpression, as analyzed by the flow cytometry assay, which indicated that miR-18a expression impaired the changes in cell proportions pre-IR and post-IR treatment to a lesser extent than the control cells (Figure 1C). Moreover, the percentage of BrdUrd- incorporating cells significantly decreased in NC-transfected cells after IR treatment, whereas no obvious alteration in the percentage of BrdUrd-incorporating cells in both breast cancer cell lines transfected with miR-18a pre-IR and post-IR treatment (Figure 1D), suggesting that miR-18a might contribute to conquering the DNA synthesis block induced by IR and enhance the radioresistant DNA synthesis of cells.

### miR-18a Suppresses ATM Expression by Targeting 3′-UTR of ATM

**Figure 2:**
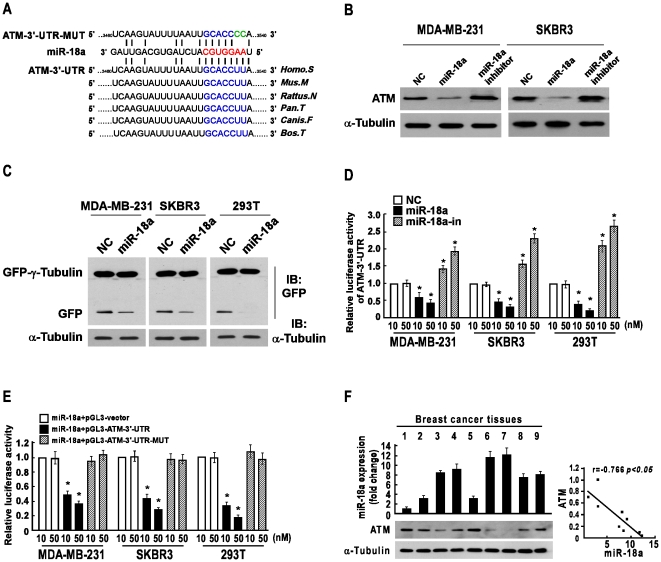
miR-18a downregulated ATM expression via directly targeting the ATM 3′-UTR. **A,** Predicted miR-18a target sequence in 3′UTR of ATM (ATM-3′-UTR) and a mutant that contained two mutated nucleotides in the ATM 3′-UTR (ATM-3′-UTR -MUT). **B,** Western blotting analysis of ATM expression in indicated cells transfected with miR-18a mimic- or miR-18a inhibitor-oligonucleotides. **C,** Western blot analysis that showed GFP expression in indicated cells. α-Tubulin was used as the loading control. **D,** Luciferase assay on indicated cells that were transduced with the pGL3-ATM-3′UTR reporter with increasing amounts (10, 50 nM) of miR-18a mimic- or miR-18a inhibitor-oligonucleotides. **E,** Luciferase assay on indicated cells transduced with pGL3-ATM-3′UTR or pGL3-ATM-3′UTR-MUT reporter with increasing amounts (10, 50 nM) of miR-18a mimic-oligonucleotides. **F,** The expression (left) and correlation (right) of miR-18a and ATM in nine freshly prepared human breast cancer tissues. Error bars represent the mean ± SD from three independent experiments. * *P*<0.05.

To investigate the abrogation mechanism of miR-18a on DNA damage induced-cell cycle arrest, we then sought to identify the molecular targets of miR-18a, which might be associated with DNA damage response. Analysis with the use of two publicly available algorithms (TargetScan and miRanda) indicated that ATM protein kinase, which has been demonstrated to be associated with the regulation of DNA damage-induced cell cycle checkpoints, is theoretically the target gene of miR-18a (Figure 2A). As expected, western blotting analysis revealed that the level of ATM protein was significantly downregulated upon miR-18a transfection but upregulated when miR-18a was suppressed (Figure 2B). Furthermore, the ATM 3̀-untranslated region (3′-UTR) fragment containing the miR-18a binding site (RE) was subcloned into the pEGFP-C3 and pGL3 luciferase reporter vectors. As shown in Figure 2C, overexpression of miR-18a in both MDA-MB-231 and SKBR3 breast cancer cell lines drastically suppressed the GFP protein expression, but not the expression of GFP-γ-tubulin that was used as the transfection control, suggesting that miR-18a specifically affected the ATM-3′-UTR. Moreover, a consistent and dose-dependent reduction of luciferase activity was observed upon miR-18a transfection in both breast cancer cell lines (Figure 2D). However, point mutations in the tentative miR-18a-binding seed region in the ATM 3′-UTR abrogated the suppressive effect of ATM expression mediated by miR-18a (Figure 2E). Thus our results demonstrate that ATM is a *bona fide* target of miR-18a.

To further examine whether the conclusions above could be supported by observations in human primary tumors, the expression levels of miR-18a and ATM were examined in 9 freshly prepared human breast tumor tissues. As shown in Figure 2F, statistical analysis demonstrated that miR-18a expression inversely correlated with ATM (r = −0.766, P<0.05). Furthermore, this inverse correlation was also observed in breast cancer cell lines (r  = −0.712, P<0.05), as indicated in Figure 1A and Figure S1. Collectively, these results further supported the notion that ATM is the target of miR-18a.

### miR-18a Impaired the ATM Signaling Pathway

**Figure 3:**
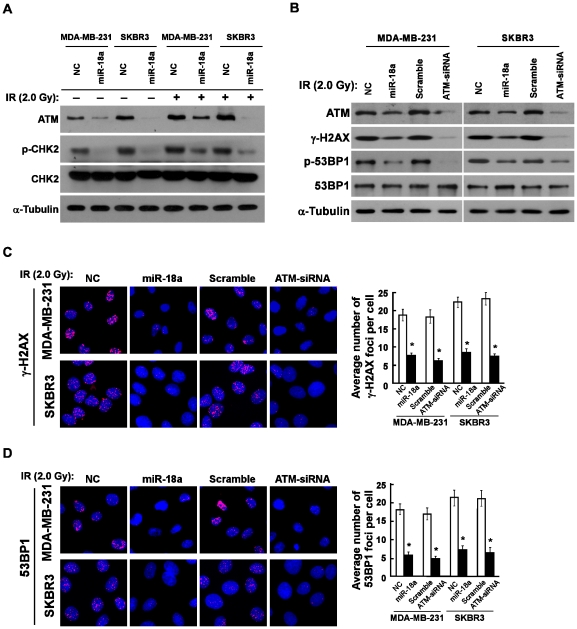
miR-18a impaired the ATM signaling pathway. **A,** Western blotting analysis of ATM expression, phosphorylated CHK2 (p-CHK2) and total CHK2 protein in indicated cells treated with or without IR. α-Tubulin was used as the loading control. **B,** Western blotting analysis of the expression levels of γ-H2AX, phosphorylated 53BP1 (p-53BP1) and total 53BP1 protein in indicated cells treated with IR (2.0 Gy). α-Tubulin was used as the loading control. **C and D,** The representative pictures (left panel) and quantification (right panel) of IR (2.0 Gy)-induced γ-H2AX (**C**) and 53BP1 (**D**) foci were analyzed in indicated breast cancer cells transfected with NC, miR-18a or scramble ATM-siRNA. The foci numbers of at least 300 cells were counted. Error bars represent the mean ± SD from three independent experiments. * *P*<0.05.

It has been reported that IR treatment could upregulate ATM expression and consequentially lead to phosphorylation of checkpoint kinase 2 (CHK2), H2AX and 53BP1 [7]–[10], [25], which promoted us to further examine the effect of miR-18a on the ATM downstream target proteins. As shown in Figure 3A, the increased expression levels of ATM protein and the phosphorylation level of CHK2 after IR treatment in both breast cancer cell lines were dramatically abolished by the transfection of miR-18a. Furthermore, Western blotting analysis revealed that ectopic expression of miR-18a drastically decreased the phosphorylation levels of H2AX (γ-H2AX) and 53BP1 (p-53BP1), which was similar to the effects of ATM depletion (Figure 3B). This was further confirmed by an indirect immunofluorescence assay, which indicated that the number of nuclear foci of H2AX and 53BP1 were significantly reduced in cells that overexpressed miR-18a (Figure 3C and 3D). Collectively, our results suggested that miR-18a impaired the ATM-mediated DNA damage signal.

### miR-18a Reduces Cell HRR Frequency and Enhances Cell Radiation Sensitivity

**Figure 4:**
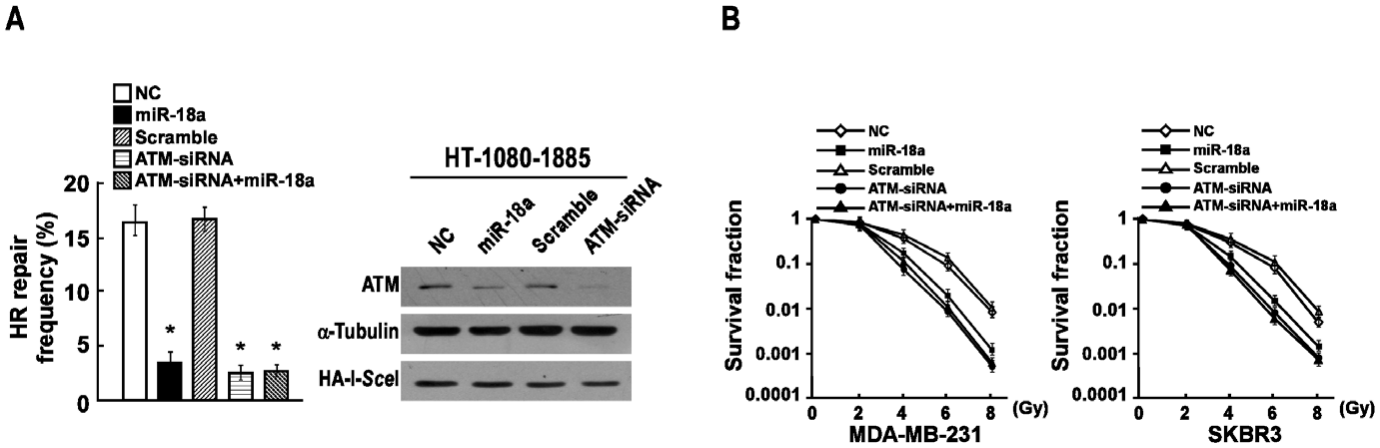
miR-18a reduces cell HRR frequency and enhances cell radiation sensitivity. **A,** DSB-induced HRR assay indicated that the overexpression of miR-18a decreased the spontaneous HR frequency in HT1080-1885 cells. Western blotting analysis of the expression levels of ATM and HA-tagged I-*Sce*I endonuclease. α-Tubulin was used as the loading control. **B,** Overexpressing miR-18a or silencing ATM expression enhanced the sensitivity of breast cancer cells to IR treatment. But further overexpressing miR-18a mimic had no additional sensitivity to IR in the ATM-silenced cells. The viabilities of the indicated cells were assayed after indicated doses of γ-radiation by the clonogenic cell survival assay. Error bars represent the mean ± SD from three independent experiments. * *P*<0.05.

Since it has been demonstrated that ATM plays an important role in the HRR [26], a DSB-induced HRR assay was performed. As shown in Figure 4A, similar to the effect of ATM depletion, overexpressing miR-18a significantly reduced HRR frequency. However, co-transfection of ATM-targeted siRNA and miR-18a did not further augment the inhibitory effect of ATM silencing on HRR (Figure 4A). As HR deficiency could sensitize cells to IR, we speculated that miR-18a expression would impact cellular sensitivity to IR treatment. As expected, a clonogenic assay showed that the overexpression of miR-18a resulted in rendering both MDA-MB-231 and SKBR3 breast cancer cell lines significantly hypersensitive to IR, but further ectopic expression of miR-18a in ATM-depleted cells did not increase sensitivity to IR (Figure 4B). These results suggest that ATM repression is essential for the role of miR-18a in HRR and cell radiation sensitivity.

To further investigate the biological role of miR-18a in the progression of breast cancer, we examine the effect of miR-18a on the ATM expression in primary normal breast epithelial cells (NBECs). As shown in Figure S2A, ectopic expression of miR-18 in NBECs dramatically decreased the ATM expression. Furthermore, western blotting analysis revealed that, in response to IR treatment, the expression levels of p-CHK2, H2AX (γ-H2AX) and p-53BP1 in the miR-18a transfected NBECs were much lower that those in vector transfected control cells (Figure S2B). Moreover, a clonogenic assay showed that overexpression of miR-18a renders NBECs hypersensitive to IR (Figure S2C). Taken together, our results suggest that upregulation of miR18a in NBECs is sufficient to impair ATM activation and HRR events upon IR treatment.

### Inhibition of miR-18a Increased ATM Expression and Reduced Cell Sensitivity to Irradiation

**Figure 5:**
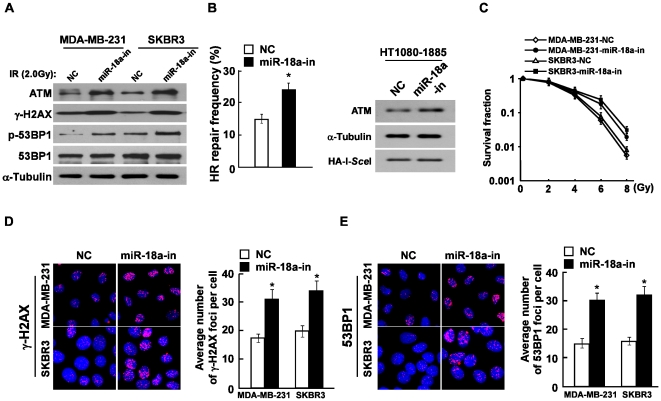
Inhibition of miR-18a increased cell HRR frequency and reduced radiosensitivity. **A,** Western blotting analysis of the expression of ATM, γ-H2AX, phosphorylated 53BP1 (p-53BP1) and total 53BP1 protein in indicated cells treated with IR (2.0 Gy). α-Tubulin was used as the loading control. **B,** DSB-induced HRR assay indicated that inhibition of miR-18a increased the spontaneous HR frequency in HT1080–1885 cells. Western blotting analysis of expression levels of ATM and HA-tagged I-*Sce*I endonuclease. α-Tubulin was used as the loading control. **C,** Inhibition of miR-18a reduced the sensitivity of breast cancer cells to IR treatment. The viabilities of the indicated cells were assayed after indicated doses of γ-radiation by the clonogenic cell survival assay. **D and E,** The representative pictures (left panel) and quantification (right panel) of IR (2.0 Gy)-induced γ-H2AX **(D)** and 53BP1 **(E)** foci were analyzed in indicated breast cancer cells transfected with either the NC or miR-18a inhibitor. The foci numbers of at least 300 cells were counted.

Next, we examined the effect of inhibiting miR-18a on the ATM expression and HRR. As shown in Figure 5A, suppression of miR-18a after the transfection of miR-18a inhibitor not only increased the expression of ATM in both breast cancer cell lines, but also dramatically increased the levels of phosphorylation levels of H2AX (γ-H2AX) and 53BP1 (p-53BP1). Furthermore, we found that the inhibition of miR-18a significantly augmented the frequency of HRR and reduced the proportion of cells that were hypersensitive to IR (Figure 5B and 5C). Moreover, the indirect immunofluorescence assay displayed that, upon IR treatment, the foci numbers of γ-H2AX and 53BP1 in the miR-18a inhibitor-transfected cells were significantly increased (Figure 5D and 5E). These results further supported the hypothesis that miR-18a impaired DNA repair through the downregulation of ATM.

## Discussion

The key finding of the current study is that miR-18a abrogated IR-induced cell cycle arrest via repression of ATM. Bioinformatic analysis and mechanistic studies revealed that miR-18a post-transcriptionally repressed ATM expression by directly targeting the ATM 3′-UTR. Furthermore, we demonstrated that, similar with the effect of ATM siRNA, ectopic expression of miR-18a impaired IR-induced DNA damage repair, enhanced radiosensitivity, and reduced the frequency of HRR. Moreover, we found that the phosphorylation levels and formation rates of nuclear foci of H2AX and 53BP1, the downstream substrates of ATM kinase, were significantly deceased in miR-18a overexpressing cells. Taken together, our results suggest that miR-18a may play an important role in the regulation of ATM.

In mammalian cells, the most serious form of DNA damage is double strand breaks, which act as potent triggers of DNA damage response [27]. This type of DNA damage can arise from exogenous agents, such as ionizing radiation (IR) and certain genotoxic chemicals and endogenous stresses, including reactive oxygen species (ROS) and mechanical stress on the chromosomes [28]. To counteract the potentially deleterious effects of DNA damage, cells have evolved two distinct pathways for the repair of DSBs, known as homologous recombination (HR) and nonhomologous end joining (NHEJ) [29]–[30]. Among these two pathways, homologous recombination repair (HRR) is mainly promoted by the serine/threonine protein kinase, ataxia-telangiectasia mutated (ATM), whose activity loss would result in low efficiency of HR-mediated DSBs repair [31]-[32].

Previously, a number of studies have reported on the mechanisms of regulation of ATM expression. It has been shown that, upon DNA damage treatment, E2F-1 directly targeted ATM promoter and elevated the expression of ATM, accompanying by an E2F-induced increase in p53 phosphorylation [25]. It has been also found that aberrant methylation of multiple CpG dinucleotides within the proximal promoter region of the ATM gene results in downregulation of ATM expression and cellular hyper-radiosensitivity [33]-[34], suggesting methylation of ATM promoter might be an alternative mechanism for downregulation of ATM. In the current study, through public available algorithms (TargetScan and miRanda), we found that ATM protein kinase, is theoretically the target gene of miR-18a. This was further determined by three different methods. Western blotting analysis showed that overexpression of miR-18a resulted in the decrease of ATM protein and sequential downregulation of phosphorylation level of ATM downstream genes, including CHK2, H2AX and 53BP1. Nuclear foci staining of γ-H2AX and 53BP1 revealed that the recruitment of ATM downstream effectors to DNA lesions was decreased by miR-18a overexpession. The luciferase activity assay and point mutation analysis demonstrated that the downregulation of ATM was mediated by miR-18a through the ATM-3′-UTR. Collectively, our results uncover a new regulatory mechanism of ATM expression in breast cancer.

miR-18a belongs to a large miR cluster oncomir-1, also called the miR-17∼92 cluster, which encodes total 5 miRNAs, including miR-17, miR-19a, miR-20a, miR-19b and miR-92a. miR17-92 cluster was found to be upregulated in lymphomas and in multiple type of solid tumors, such as breast, colon, lung, stomach, and pancreas cancers [35]–[36]. The elevated expression of miR17-92 gene cluster could also be genome-caused as the miR17-92 gene cluster genome locus was found to be amplified in hematopoietic malignancies [37]. Meanwhile, it has been reported that miR17–92 gene cluster transcript could be activated by c-myc, N-myc and E2F families [38]–[40]. Interestingly, the expression levels of each miRNA in the cluster is not exactly parallel with each other, suggesting that processing or stability of the miRNAs is differentially regulated [41]. Guil and colleagues have found that RNA-binding protein hnRNP Al, which is overexpressed in breast cancer, could facilitate Drosha-mediated processing of miR-18a [42]–[43]. Previously, several studies have demonstrated that miR-17∼92 cluster plays important role in the progression and development of breast cancer. It has been shown that overexpression of miR-17 promotes human breast cancer cell migration and invasion through downregulation of HBP1 [44]. Suppression of miR-17 and miR-20a could inhibit growth and invasion of breast cancer cells via upregulation of tumor suppressor ZBTB4 [45]. Al-Nakhle et al reported that overexpressing miR-19 in breast cancer cells resulted in downregulation of ERbeta1 via direct targeting of its 3′-UTR [46]. In the current study, we demonstrated that miR-18a reduced DNA damage repair signaling and increased cellular radio-sensitivity by repression of ATM, which further support the notion that miR17–92 cluster functions as oncomir.

In summary, the current study provides, for the first time, an important link between miR-18a-impaired DNA damage response and downregulation of ATM in breast cancer. Our findings revealed the significance of miR-18a in regulating cell cycle checkpoints, DSBs repair and radiosensitivity. Understanding the precise role played by miR-18a in DNA damage response processes will not only increase our knowledge of breast cancer progression but miR-18a might also be a signature of the DNA damage response decision and a potential therapeutic target.

## Materials and Methods

### Ethics Statement

This study was conducted on a total of 19 breast cancer samples that were histopathologically and clinically diagnosed at the Sun Yat-sen University Cancer Center from 2009 to 2010. For the use of clinical materials for research purposes, prior patients' consents and approval were obtained from the Sun Yat-sen University and Cancer Center Institutional Board. All samples were collected and analyzed with prior written informed consents from the patients.

Primary NBEC was isolated from the mammoplasty material of a 30-year-old woman and approved by the Sun Yat-sen University and First Affiliated Hospital Institutional Board. Sample was collected and analyzed with written informed consent. Primary NBEC was cultured in the Keratinocyte serum-free medium (Invitrogen, Carlsbad, CA) supplemented with epithelial growth factor, bovine pituitary extract and antibiotics (120 µg/ml streptomycin and 120 µg/ml penicillin). Breast cancer cell lines, MDA-MB-231 and SKBR3 were grown in DMEM medium (Invitrogen, Carlsbad, CA) su with 10% fetal bovine serum (FBS) (HyClone, Logan, UT) and 100 units of penicillin-streptomycin. Cells were maintained and experiments were accomplished in a humidified 37°C with 5% CO_2_.

### RNA Extraction, Reverse Transcription (RT) and Real-time PCR

Total miRNA from cultured cells and fresh surgical breast cancer tissues was extracted using the mirVana miRNA Isolation Kit (Ambion, Austin, TX, USA) according to the manufacturer's instructions. cDNA was synthesized from 5 ng of total RNA using the Taqman miRNA reverse transcription kit (Applied Biosystems, Foster City, CA), and the expression levels of miR-18a were quantified using miRNA-specific TaqMan MiRNA Assay Kit (Applied Biosystems). Real-time PCR was performed using the Applied Biosystems 7500 Sequence Detection system. The expression of miR-18a was defined based on the threshold cycle (Ct), and relative expression levels were calculated as 2^−[(Ct of miR−18a) − (Ct^ ^of *U6*)]^ after normalization with reference to expression of U6 small nuclear RNA.

### Western Blotting

Western blotting was performed according to standard methods as described previously [47], using anti-ATM, anti-CHK2, and anti-p-CHK2 antibodies, anti-53BP1, anti-p-53BP1 (Cell Signaling, Danvers, MA), anti-γ-H2AX (Abcam, Cambridge, MA), anti-HA (Sigma, Saint Louis, MI). The membranes were stripped and re-probed with an anti-α-tubulin antibody (Sigma, Saint Louis, MI) as a loading control.

### Plasmids, siRNA and Transfection

The region of human ATM 3′-UTR, from 3340 to 3540, generated by PCR amplification from DNA of the SKBR3 cells, was cloned into pEGFP-C1 (Clontech, Mountain View, CA) and pGL3 vector (Promega, Madison, WI). The primers selected were as the following: ATM-3′UTR-GFP-up:GCCAGATCTTGAGAAATATAGAGATGTG;ATM-3′UTR-GFP-dn:GCCGAATTCGCTTTTAGAATTATT;ATM-3′UTR-luc-up:GCCCCGCGGGAAATATAGAGATGTG;ATM-3′UTR-luc-dn:GCCCTGCAGGCTTTTAGAATTATT;ATM-3′UTR-MUT-luc-up:GTATTTTAATTGCACCTTAATGAAATTATCTATT;ATM-3′UTR-MUT-luc-dn:AATAGATAATTTCATTAAGGTGCAATTAAAATAC. For depletion of ATM, the siRNA was synthesized and purified by Ribobio Inc. (Guangzhou, Guangdong, China). The ATM siRNA sequence used was: TGGTGCTATTTACGGAGCT. Transfection of siRNAs or plasmids was performed using the Lipofectamine 2000 reagent (Invitrogen) according to the manufacturer's instruction.

### Luciferase Assay

Cells (3.5×10^4^) were seeded in triplicates in 48-well plates and allowed to settle for 24 h. One hundred nanogram of luciferase reporter plasmids or the control-luciferase plasmid, plus 10 ng of pRL-TK renilla plasmid (Promega, Madison, WI), were transfected into cells using the Lipofectamine 2000 reagent (Invitrogen Co., Carlsbad, CA) according to the manufacturer's recommendation. Luciferase and renilla signals were measured 48 h after transfection using the Dual Luciferase Reporter Assay Kit (Promega, Madison, WI) according to a protocol provided by the manufacturer. Three independent experiments were performed and the data are presented as mean ± SD.

### Bromodeoxyuridine Labeling (BrdUrd Incorporation Assay)

To analyze the S-phase checkpoint, cells grown on coverslips (Fisher Scientific) at 70% confluence were irradiated with the indicated dose and incubated for 20 h. BrdUrd was added to cells and incubated for 2 h. Cells were then fixed and stained with anti-BrdUrd antibody (Upstate, Temecula, CA) according to the manufacturer's instructions. Gray level images were acquired under a laser scanning microscope (Axioskop 2 plus, Carl Zeiss Co. Ltd., Jena, Germany).

### Immuno fluorescence

Cells grown on coverslips (Fisher Scientific) were fixed in ice-cold methanol for 10 min, blocked with 10% goat serum in PBS, and incubated with anti-γ-H2AX, anti-53BP1, in 10% goat serum/PBS. The primary antibodies were detected with rhodamine-conjugated goat anti-rabbit IgG (The Jackson Laboratory) and the DNA was stained with DAPI. Gray level images were acquired under a laser scanning microscope (Axioskop 2 plus, Carl Zeiss Co. Ltd., Jena, Germany).

### Flow Cytometry Analysis

Cells were irradiated with the indicated dose and incubated for 20 h. All cells were then harvested by trypsinization, washed in ice-cold PBS, and fixed in 80% ice-cold ethanol in PBS. Before staining, the cells were spun down in a cooled centrifuge and resuspended in the cold. Bovine pancreatic RNAase (Sigma-Aldrich) was added at a final concentration of 2 µg/ml, and cells were incubated at 37°C for 30 min, followed by incubation in 20 µg/ml of propidium iodide (Sigma-Aldrich) for 20 min at room temperature. 50,000 cells were analyzed on a flow cytometer (FACSCalibur; BD Biosciences).

### Clonogenic Survival Assay

Cells were plated at 500 cells per well onto a six-well dish in triplicate and then incubated for 24 h to allow settling. Cells were treated with a series of IR dose (0, 2, 4, 6, 8Gy). Clonogenic Survival Assay was performed according to the standard methods described in [48].

### DSB-induced HRR Assay

HRR was assayed essentially as described previously [49]. Briefly, HT-1080–1885 cells were transfected with miR-18a mimic or negative control mimic anlong with pCMV(HA-3xNLS)I-SceI that expresses a HA epitope- and nuclear localization signal-tagged I-SceI nuclease. Forty-eight hours later, ∼100,000 viable cells were transferred to 10-cm-diameter dishes, and HRR products were selected in growth medium with 325 µg of G418/ml. G418-resistant colonies were scored 12 to 14 days later. The plating efficiency was determined by seeding the appropriate numbers of cells to 10-cm-diameter dishes with growth medium lacking G418. HRR frequencies were calculated as the number of G418-resistant colonies per viable cell plated in G418 medium. Background HR levels were estimated in parallel experiments, except that cells were transfected with an empty pCMV vector.

## Supporting Information

Figure S1 **ATM expression in breast cancer cells and inverse correlation between miR-18a expression and ATM expression.** Western blotting analysis of ATM expression (left) and correlation (right) of miR-18a expression and ATM expression in normal breast epithelial cells (NBEC) and breast cancer cell lines, including ZR-75-1, ZR-75-30, SKBR3, T47D, MDA-MB-231, MDA-MB-435, MDA-MB-453, BT474 and BT-549.
(TIF)

Figure S2 **Overexpression of miR18a is sufficient to impair ATM activation and HRR events in response to IR in NBECs. A,** Western blotting analysis of the expression of ATM in NBECs transfected with NC or miR-18a. α-Tubulin was used as the loading control. **B,** Western blotting analysis of the expression phosphorylated CHK2 (p-CHK2), total CHK2, phosphorylated 53BP1 (p-53BP1), total 53BP1 and γ-H2AX protein in NBECs in response to IR (2.0 Gy) treatment. α-Tubulin was used as the loading control. **C,** Overexpression of miR-18a increased the sensitivity of NBECs to IR treatment. The viabilities of the indicated cells were assayed after indicated doses of γ-radiation by the clonogenic cell survival assay.
(TIF)
