# Supplementary figures and images for: miR-18a Impairs DNA Damage Response through Downregulation of Ataxia Telangiectasia Mutated (ATM) Kinase
Source: PLoS One. 2011 Sep 27;6(9):e25454. doi: 10.1371/journal.pone.0025454 (PMC3181320; doi:10.1371/journal.pone.0025454)

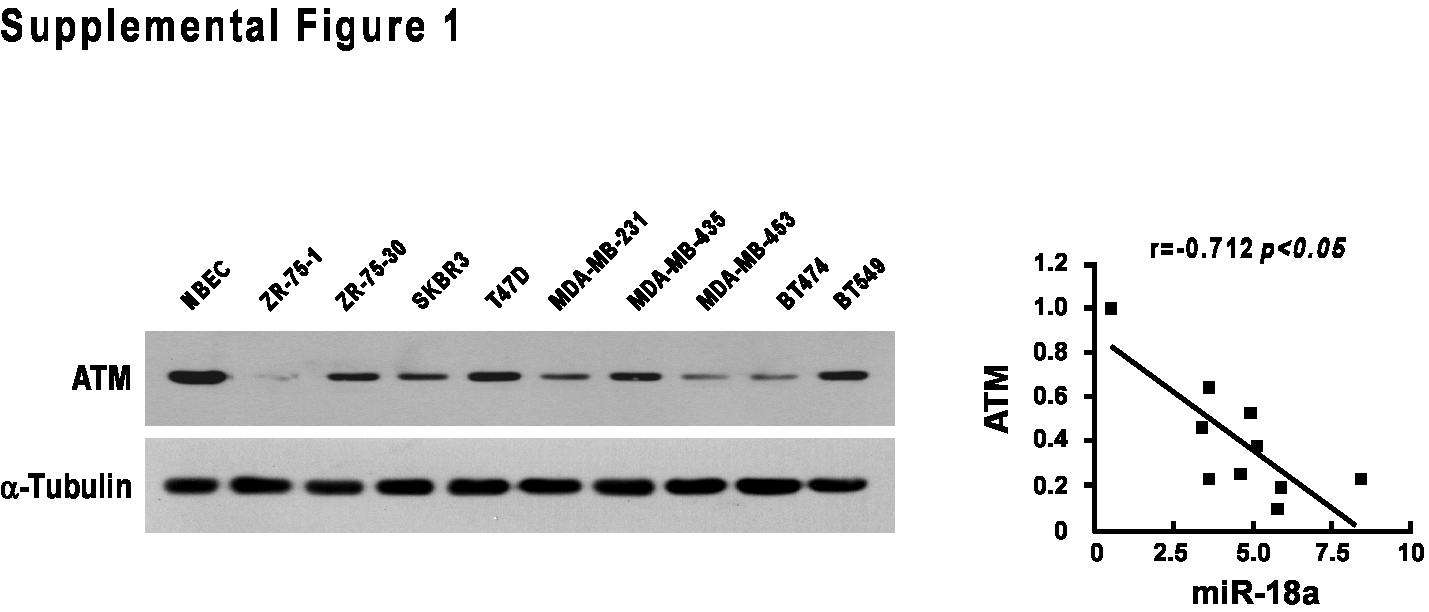

Supplement: Figure S1 — ATM expression in breast cancer cells and inverse correlation between miR-18a expression and ATM expression. Western blotting analysis of ATM expression (left) and correlation (right) of miR-18a expression and ATM expression in normal breast epithelial cells (NBEC) and breast cancer cell lines, including ZR-75-1, ZR-75-30, SKBR3, T47D, MDA-MB-231, MDA-MB-435, MDA-MB-453, BT474 and BT-549. (TIF) [file pone.0025454.s001.tif]

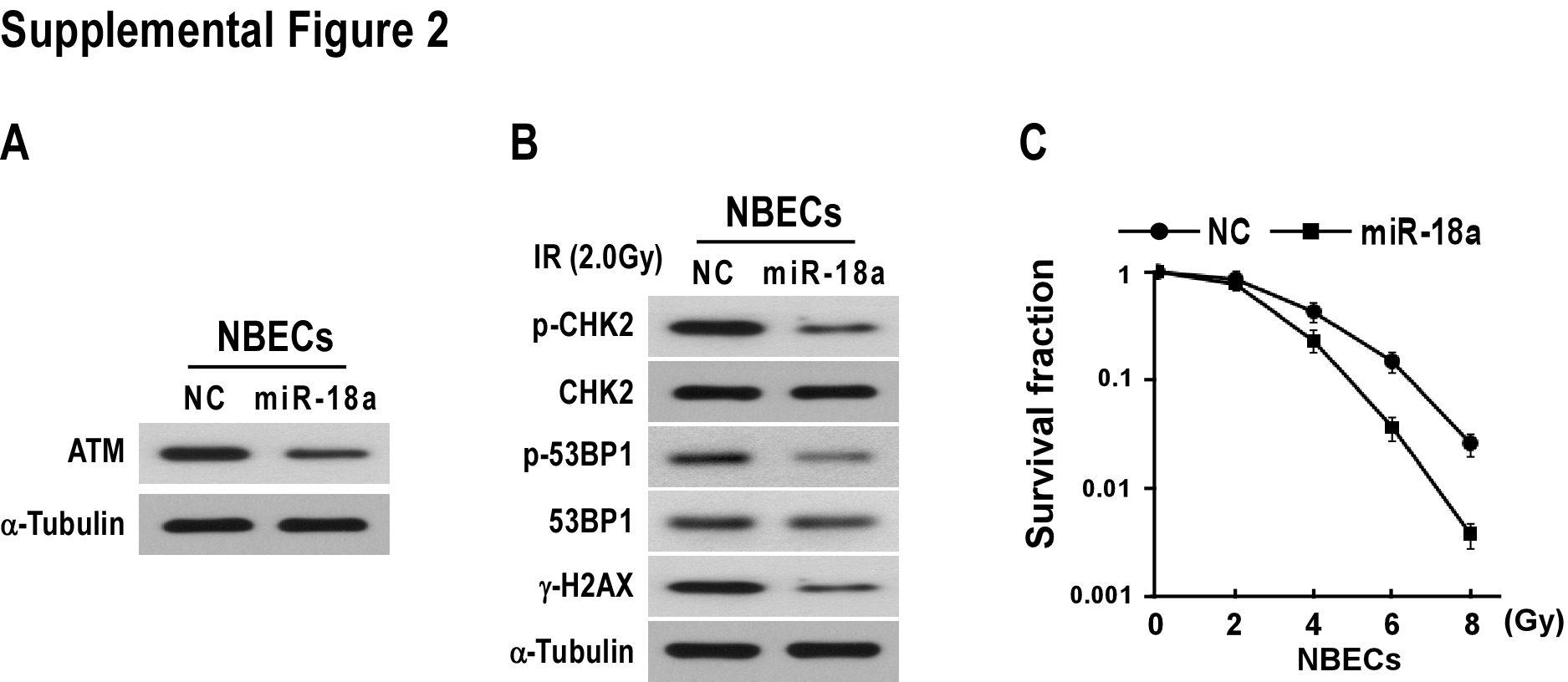

Supplement: Figure S2 — Overexpression of miR18a is sufficient to impair ATM activation and HRR events in response to IR in NBECs. A, Western blotting analysis of the expression of ATM in NBECs transfected with NC or miR-18a. α-Tubulin was used as the loading control. B, Western blotting analysis of the expression phosphorylated CHK2 (p-CHK2), total CHK2, phosphorylated 53BP1 (p-53BP1), total 53BP1 and γ-H2AX protein in NBECs in response to IR (2.0 Gy) treatment. α-Tubulin was used as the loading control. C, Overexpression of miR-18a increased the sensitivity of NBECs to IR treatment. The viabilities of the indicated cells were assayed after indicated doses of γ-radiation by the clonogenic cell survival assay. (TIF) [file pone.0025454.s002.tif]
